# Ursodeoxycholic Acid at 18–22 mg/kg/d Showed a Promising Capacity for Treating Refractory Primary Biliary Cholangitis

**DOI:** 10.1155/2021/6691425

**Published:** 2021-01-21

**Authors:** Xinyu Xiang, Xiaoli Yang, Mengyi Shen, Chen Huang, Yifeng Liu, Xiaoli Fan, Li Yang

**Affiliations:** ^1^Department of Gastroenterology and Hepatology, Sichuan University-University of Oxford Huaxi Joint Centre for Gastrointestinal Cancer, West China Hospital, Sichuan University, Chengdu, China; ^2^Department of Gastroenterology & Hepatology, West China Hospital, Sichuan University, Chengdu, Sichuan 610041, China

## Abstract

**Aim:**

To compare the response between the current recommended dosage 13–15 mg/kg/d and 20 mg/kg/d dose of ursodeoxycholic acid (UDCA) in primary biliary cholangitis (PBC) patients who do not respond completely to a standard dose of UDCA.

**Methods:**

We included 73 patients with poor response and randomized them into two groups to investigate whether increasing the dosage of UDCA was beneficial to nonresponders. Patients assigned to the 13–15 mg/kg/d group continued with standard therapy, and participants in the 18–22 mg/kg/d group switched to the higher dosage (18–22 mg/kg/d), with a follow-up of 12 months for both groups. The primary endpoints were the rate of response at 6 months and drug side effects.

**Results:**

According to the Paris 2 criteria, patients receiving 18–22 mg/kg/d UDCA achieved a response rate of 59.4% compared with 36.1% in the standard dosage group (*P*=0.046) at 6 months, respectively. At 12 months, the high-UDCA-dosage group achieved a response rate of 59.4% compared with 47.2% in the standard dosage group (*P*=0.295), respectively. Additionally, the risk score predicted by the UK-PBC model was lower in high-dosage UDCA-treated patients than in the standard dosage group (all *P* < 0.05). Side effects include diarrhea, nausea and vomiting, rash, and newly developed high blood pressure, which were mild and tolerated.

**Conclusions:**

Patients treated with the high UDCA dosage showed some advantages over those who continued the standard dosage in terms of biochemical remission and disease progression, indicating that standard therapy with UDCA for 6 months and then another 1 year with high UDCA dosage for nonresponders could be a treatment option before second-line therapy is recommended.

## 1. Introduction

Primary biliary cholangitis (PBC), an autoimmune disease predominantly affecting females, is characterized by clinical manifestations of progressive cholestasis, chronic elevation of alkaline phosphatase (ALP), and positive antimitochondrial antibodies (AMAs) [1]. Liver biopsy typically presents as nonsuppurative destructive cholangitis, and histological evidence is required only when antibodies are absent [2, 3]. Untreated patients can develop cirrhosis, hepatic failure, and liver-related death, and lifelong medication use is strongly recommended [4].

Ursodeoxycholic acid (UDCA) has been recommended as the first-line therapy for PBC patients according to guidelines since its approval by the Food and Drug Administration (FDA) in 1977, as it benefits biochemical mitigation and transplantation-free survival [5, 6]. UDCA was also found to slow the patient's histological progress compared with a placebo [7]. Studies have revealed quantitative and compositional changes in the bile pool of PBC patients, and a positive correlation was found between the response and enrichment of UDCA in the patient's bile pool [8].

Despite the efficacy, it was estimated that nearly 40% of PBC patients on standard UDCA therapy did not achieve a complete response [9]. Many clinical trials have been carried out to explore alternative options for those nonresponders, one of which was an increased dosage of UDCA. A prior clinical trial comparing the effectiveness between 20 mg/kg/d and 10 mg/kg/d UDCA found that the 20 mg/kg/d dosage resulted in greater biochemical decreases [10], yet no trials were conducted to compare the response between the current recommended dosage 13–15 mg/kg/d and 20 mg/kg/d. Therefore, we performed this study to investigate the efficacy and safety of UDCA at a dosage of 18–22 mg/kg/d in treating PBC patients with a poor response.

## 2. Methods

### 2.1. Study Population

Patients with a recent or previous diagnosis of PBC were recruited at the inpatient and outpatient clinic of the Gastroenterology and Hepatology Department, West China Hospital. PBC was diagnosed when at least two of the following three criteria were fulfilled: (1) serum alkaline phosphatase (ALP) levels at least 1.5 times the normal upper limit for more than 6 months; (2) the presence of AMAs in serum; and (3) representative histological manifestations of portal area inflammation and bile duct injury [11]. After 6 months of standard treatment, patients with an incomplete response, which was defined as the Paris 2 criteria [12] (a serum level of ALP or aspartate aminotransferase (AST) > 1.5 times the upper limit of the normal range or an abnormal total bilirubin (TB) level), were eligible for entry. The inclusion criteria were patients diagnosed with PBC who were treated with 13–15 mg/kg/d UDCA at West China Hospital for 6 months and obtained a suboptimal response to UDCA. The exclusion criteria included diseases that are easily confused with PBC, e.g., autoimmune hepatitis and primary sclerosing cholangitis. Cirrhosis was evaluated according to the liver histology, abdominal imaging, such as ultrasonography (US), computed tomography (CT), and/or magnetic resonance imaging (MRI), or endoscopic examination [13]. Patients with decompensated cirrhosis were excluded, which was diagnosed by the presence of clinical complications [14].

Patients underwent autoimmune investigations: AMA, anti-Sp100 and anti-gp210 testing by immunoblotting (IB), and indirect immunofluorescence, searching for PBC-specific ANAs. All parameters were examined in the Department of Laboratory Medicine of West China Hospital, which was certified by the College of American Pathologists (CAP). Some cases underwent investigations for anti-Sp100 and anti-gp210, which were carried out by the third party (Kindstar Global Corporation, China).

### 2.2. Study Design

For this study, we conducted a prospective, randomized, open-label pilot trial to investigate whether a higher dosage of UDCA therapy could benefit PBC patients and per-protocol analysis was applied. All recruited patients were given 13–15 mg/kg/d UDCA at the time of diagnosis according to the current guidelines [15]. They were required to complete a serum biochemical blood test at 6 months. Patients who did not meet the suggested response of the Paris 2 criteria were considered nonresponders. After confirmation of eligibility, we randomly allocated participants to receive a higher dosage of UDCA (18–22 mg/kg/d) or to continue with the previous 13–15 mg/kg/d UDCA therapy. The drug side effects observed included diarrhea, nausea and vomiting, rash, and new onset of high blood pressure [16]. The response was evaluated in the two groups at 6 months and 12 months. Prognostic evaluation was determined by the established UK-PBC score and Globe score [17].

Assuming a dropout rate of 10%, it was estimated that a sample of 50 patients was needed with a projected response rate of 50% in the high-dosage group and 10% in the standard group to have 90% power with a significant difference at a value of *α* = 0.05. This study was registered at ClinicalTrials.gov (identifier: NCT03345589), and all patients provided written informed consent.

### 2.3. Outcomes

The primary outcome was the percentage of patients with a complete biochemical response as defined by the Paris 2 criteria and side effects. The UK-PBC scoring system was used to detect the anticipated 5-, 10-, and 15-year risks of liver transplantation, and liver-related fatality and Globe scores were determined to predict transplant-free survival. The UK-PBC score incorporates the baseline platelet count and serum albumin level and the serum bilirubin, transaminases, and ALP levels measured after 12 months of UDCA treatment [17]. Secondary outcomes included changes from baseline in serum TB, ALP, AST, alanine aminotransferase, *γ*-glutamyltransferase (GGT), albumin, globulin, immunoglobulin M, and so on; liver fibrosis was measured by noninvasive indices, including the AST-to-platelet ratio index (APRI) and the fibrosis index based on four factors (FIB-4). The formula used for calculating FIB-4 equates to age (years)xAST (U/L)/(platelets [10^9^/L]x(ALT [U/L])^1/2^) [18]; we also compared the response difference by definition of the Barcelona criteria because patients had been on medication at 6 months and 1 year (Barcelona criteria: ALP decrease >40% of pretreatment levels or normalization), respectively.

### 2.4. Statistical Analysis

A chi-square test was performed to compare the differences in the percentage of patients with a complete biochemical response in the two groups. Comparisons of baseline data, UK-PBC scores, APRI, and FIB-4 values were performed using the Mann–Whitney *U* test. Continuous variables are expressed as medians with interquartile range. Two-sided *P* values of less than 0.05 were considered statistically significant. The statistical analysis was performed using SPSS 24.0 (SPSS version 24.0 for Windows; IBM Corp, Armonk, NY, USA).

## 3. Results

**Figure 1:**
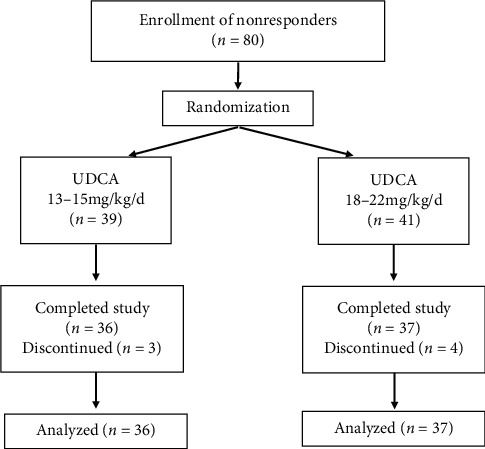
Flowchart of the trial.

Our trial was conducted following the procedure steps demonstrated in Figure 1. Patients at entry exhibited no differences in serum biochemical markers (Table 1). More than 90% of the population in both groups consisted of females in their 50s. No differences were found in the percentage of fatigue and pruritus when enrolled in the study (35.1% vs. 30.6% and 51.4% vs. 50.0%, respectively). 61.1% of the population in the standard group and 78.3% in the high-dosage group was AMA positive. Multiple nuclear dot (MND) and rim-like/membranous antinuclear (RL/M) antibodies were rare findings in our study group, and only 12 cases in our study underwent investigations for anti-Sp100 and anti-gp210 by immunoblotting. No differences were found in assessments of degree of fibrosis according to the APRI and FIB-4 values and the percentage of cirrhosis between the standard and high-dosage groups at baseline.

At 6 months, response rates assessed by the Paris 2 criteria were significantly different between the two groups, with 59.4% of patients achieving a complete response in the high-dosage group compared with 36.1% in the standard group (*P*=0.046). In contrast, under the Barcelona criteria, the high-dosage group did not show a significant increase in the proportion of responders (*P*=0.483) (Table 2). At 12 months, there was no difference in the response rates between the two groups using Paris 2 and Barcelona criteria, respectively (Table 3). The UK-PBC score at 12 months showed a lower incidence of severe liver-related events in the 18–22 mg group than in the group continuing with the standard UDCA dosage (Table 4). APRI and FIB-4 scores of high-dosage patients were lower than that of the standard dosage group at 6 and 12 months (all *P* < 0.05) (Table 5).

Overall, changes from baseline in the levels of critical biomarkers of PBC were partly consistent with the primary outcome. The reduction rate of ALB and PLT in the high-dosage group was higher than that of the standard group at 6 months (Table 6). However, the reduction rate of the levels of these critical biomarkers was similar in both groups at 12 months (Table 7).

To assess safety, side effects of UDCA, including diarrhea, nausea and vomiting, rash, and newly developed high blood pressure, were evaluated. Two patients in the high-dosage group developed mild diarrhea, which was tolerable. Generally, both dosages were well tolerated by patients with no occurrence of serious adverse reactions. More detailed information is presented in Table 8.

## 4. Discussion

The primary outcomes suggested that a dosage of 18–22 mg was superior to 13–15 mg in treating PBC nonresponders when measured by the Paris 2 criteria. It is reasonable to draw the conclusion that an 18–22 mg dosage is more effective than standard therapy in these conditions. The UK-PBC score predicted the advantage of 18–22 mg of UDCA in preventing liver-related adverse events and decreasing the risk of liver transplantation. Also, lower APRI and FIB-4 indices in high-dosage patients indicated that increasing the dosage might delay the development of hepatic cirrhosis.

PBC-specific ANAs are associated with severe PBC and their presence may also predict a less response to UDCA treatment [19, 20]. In our study population, MND and RL/M antinuclear antibodies were rare findings, which implied a favourable course. According to the Paris 2 criteria, the biochemical response was assessed a year after taking the standard dosage of UDCA. However, an early identification was found by Zhang et al. and measurements at six months may be used instead of measurements at 1 year because these values exhibited higher or the same positive predictive value (PPV) and negative predictive value (NPV) [21]. Furthermore, a study that aimed at evaluating the effect of bezafibrate compared to UDCA assessed patients after 6 months of drug treatment as well. Therefore, we determined whether a patient was a responder or nonresponder at 6 months after medications were administered [22].

In contrast to our results, one pilot study, where twice the standard dosage was tested, concluded that a double dosage may not benefit the majority of refractory conditions [23]. Nonresponders enrolled in that trial were reported to have been on standard UDCA therapy for 24–141 months [23], which is longer than our patients, and this may be one of the reasons for the difference between our conclusions. However, the administration of high-dose UDCA to early-stage PBC patients in another trial was found to be positively correlated with the UDCA concentration in bile acids (BAs) and improved liver function [24], which supported our conclusion.

With several clinical trials demonstrating the therapeutic effect of obeticholic acid (OCA) and fibrates, both drugs are now being proposed as second-line treatment options for patients who respond incompletely to or do not tolerate UDCA [25]. Approximately 50% and 30% of patients biochemically respond to OCA and fibrates, respectively, with significantly decreased ALP and normalized TB levels [22, 26]. However, side effects occurred more commonly in the treated patients than in the placebo group. Pruritus, the most common complaint of OCA, occurred in more than half of the patients on novel therapy [26]. UDCA, a natural endogenous BA, alleviates cholestasis and reduces damage to cholangiocytes primarily by promoting bile excretion and decreasing BA toxicity [9]. BAs make up an important component of bile, and some are hydrophobic in nature and have toxic effects on the bile duct epithelium. The proportion of UDCA in the bile composition of PBC patients was substantially increased, replacing the toxic BAs and acting as a cytoprotective agent [8, 27]. Interestingly, the toxicity of BAs is pH-dependent. UDCA promotes bicarbonate excretion and reduces protonation of BA so that it avoids BA-induced apoptosis [28]. From our result, we can find that adverse reactions were not observed with high-dosage UDCA, which meant the high dosage was well tolerated. Though the gain in terms of biochemical response was not impressive at 12 months, the risk score predicted by the UK-PBC model and the noninvasive scores of assessments of fibrosis may also be predictive of a favourable course. Therefore, patients treated with the high UDCA dosage showed some advantages over those who continued the standard dosage in terms of biochemical remission at 6 months and disease progression at 6 and 12 months, indicating that standard therapy with UDCA for 6 months and then another 1 year with high-dosage UDCA for nonresponders may be considered before second-line therapy is recommended.

In this trial, we measured the noninvasive biomarkers APRI and FIB-4 scores to assess the extent of liver fibrosis. The APRI and FIB-4 values have been widely used to predict fibrosis and risk of events [29, 30]. The UK-PBC scoring system comprehensively integrated relevant prognostic variables and were tested in large, multicenter-based cohorts. Their discriminative ability has been demonstrated to be superior to other existing criteria [17, 31]. Hence, it is likely that patients in the high-dosage group have less disease progression than those in the standard group.

There are still some limitations of our trial that should be noted. The clinical presentation of patients, such as fatigue and pruritus, was not considered, as fatigue and/or pruritus at onset predict a poorer response to UDCA and prognosis [32, 33]. Evidence from liver biopsy to provide support from a histological perspective was lacking. Also, there was a potential bias in the evaluation of UK-PBC scores as the presence of cirrhosis seemed to be more frequent in the standard therapy group compared with the high-dose UDCA group, though there was no statistically significant difference.

In conclusion, our study shows that increasing the dosage of UDCA contributes to the therapeutic potency for treatment of PBC nonresponders, indicating that standard therapy with UDCA for 6 months and then another 1 year with high-dosage UDCA for nonresponders could be a treatment algorithm before second-line therapy is recommended.

## Figures and Tables

**Table 1:** Characteristics of study population at entry.

	13–15 mg/kg/d	18–22 mg/kg/d	*P* value
	(*n* = 36)	(*n* = 37)	
Age, year	52.0 (46.0, 58.8)	52.0 (46.5 – 56.0)	0.623
Female, no. (%)	30 (83.3%)	33 (89.2%)	0.476
Fatigue (%)	11/36 (30.6%)	13/37 (35.1%)	0.677
Pruritus (%)	18 (50.0%)	19 (51.4%)	0.995
PLT, 10^9^/L	170.0 (102.3, 234.5)	181.0 (137.5, 235.5)	0.318
TB, *μ* mol/L	17.4 (14.1, 27.3)	15.3 (11.2, 22.4)	0.072
ALP, IU/L	281.0 (216.0, 394.5)	238.0 (203.0, 338.0)	0.156
AST, IU/L	58.5 (45.0, 94.0)	54.0 (42.5 – 67.0)	0.180
ALT, IU/L	64.0 (40.5, 77.0)	53.0 (44.0, 78.0)	0.938
ALB, g/L	43.3 (39.7, 47.5)	45.4 (43.2, 46.6)	0.133
GLB, g/L	36.7 (31.3, 39.2)	34.3 (30.6, 35.6)	0.090
GGT, IU/L	220.0 (123.3, 288.0)	161.0 (110.5 – 261.5)	0.227
IGM, mg/L	3145.0 (1787.0, 4265.0)	3000.0 (2090.0, 3815.0)	0.728
CHOL, mmol/L	5.3 (4.4, 6.9)	6.0 (5.3 – 6.4)	0.348
APRI	1.17 (0.64, 1.18)	0.97 (0.61, 1.27)	0.120
FIB-4	3.02 (1.49, 5.62)	2.14 (1.41, 3.00)	0.085
AMA, no. (%)	22 (61.1%)	29 (78.3%)	0.108
MND (%)	0 (0%)	1 (2.7%)	>0.999
Anti-Sp100*∗*	0/5 (0%)	1/7 (14.3%)	>0.999
RL/M (%)	11 (30.6%)	12 (32.4%)	0.863
Anti-gp210*∗*	1/5 (20.0%)	2/7 (28.6%)	>0.999
Liver cirrhosis, no. (%)	12 (33.3%)	7 (18.9%)	0.181

*∗*Only 12 cases in our trial underwent investigations for anti-Sp100 and anti-gp210 by immunoblotting. *Note.* PLT, platelet count; TB, total bilirubin; ALP, alkaline phosphatase; AST, aspartate aminotransferase; ALT, alanine aminotransferase; ALB, albumin; GLB, globulin; GGT, *γ*-glutamyltransferase; IGM, immunoglobulin M; CHOL, cholesterol; PT, prothrombin time; APRI, aspartate aminotransferase/platelet ratio index; FIB-4, fibrosis index based on the four factors; AMA, antimitochondrial antibody; MND, multiple nuclear dots; RL/M, rim-like/membranous.

**Table 2:** Response by definition of Barcelona and Paris-II criteria at 6 months.

6-month	Response rate	*P* value
Paris-II		
13 – 15 mg/kg/d	13/36 (36.1%)	0.046
18 – 22 mg/kg/d	22/37 (59.4%)	
Barcelona		
13 – 15 mg/kg/d	9/36 (25.0%)	0.483
18 – 22 mg/kg/d	12/37 (32.4%)	

**Table 3:** Response by definition of Barcelona and Paris-II criteria at 12 months.

12-month	Response rate	*P* value
Paris-II		
13 – 15 mg/kg/d	17/36 (47.2%)	0.295
18 – 22 mg/kg/d	22/37 (59.4%)	
Barcelona		
13 – 15 mg/kg/d	14/36 (38.9%)	0.401
18 – 22 mg/kg/d	18/37 (48.6%)	

**Table 4:** UK-PBC risk score.

	13–15 mg/kg/d (*n* = 36)	18–22 mg/kg/d (*n* = 37)	*P* value
UK-PBC-5 year	0.0257 (0.0118, 0.0614)	0.0152 (0.0100, 0.027)	0.006
UK-PBC-10 year	0.0835 (0.0391, 0.1912)	0.0533 (0.0362, 0.0809)	0.024
UK-PBC-15 year	0.1498 (0.0714, 0.3263)	0.0943 (0.0664, 0.1453)	0.025

**Table 5:** Fibrosis risk score testing at the 6th and 12th month.

	13–15 mg/kg/d	18–22 mg/kg/d	*P* value
6 month			
APRI	1.016 (0.530, 2.012)	0.615 (0.473, 1.135)	0.041
FIB-4	2.501 (1.550, 4.814)	1.729 (1.193, 2.549)	0.018
12 month			
APRI	1.165 (0.641, 2.038)	0.731 (0.467, 1.114)	0.008
FIB-4	2.874 (1.794, 4.993)	2.036 (1.343, 2.691)	0.013

*Note.* APRI, aspartate aminotransferase/platelet ratio index; FIB-4, fibrosis index based on the four factors.

**Table 6:** Percentage of biochemical relative changes from baseline at 6 months.

%Change from baseline	13–15 mg/kg/d	18–22 mg/kg/d	*P* value
	(*n* = 36)	(*n* = 37)	
TB, *μ* mol/L	6.79 (−12.31, 23.09)	9.69 (−9.91, 26.70)	0.544
ALP, IU/L	9.60 (−4.03, 28.13)	16.09 (4.02, 28.12)	0.238
GGT, IU/L	31.57 (7.83, 42.76)	−29.18 (19.22, 40.03)	0.974
AST, IU/L	10.56 (−4.80, 28.25)	15.15 (5.00, 32.67)	0.544
ALT, IU/L	10.71 (−4.27, 34.31)	21.28 (0.55, 39.00)	0.522
ALB, g/L	−2.21 (−8.37, 2.97)	1.76 (−1.89, 6.17)	0.013
GLB, g/L	2.18 (6.19, 6.70)	2.23 (−2.51, 9.94)	0.460
PLT, 10^9^/L	−9.49 (21.55, 1.59)	1.72 (−12.71, 22.83)	0.034
IGM, mg/L	8.36 (−5.45, 19.09)	6.67 (0.72, 15.04)	0.501

*Note.* TB, total bilirubin; ALP, alkaline phosphatase; AST, aspartate aminotransferase; ALT, alanine aminotransferase; ALB, albumin; GLB, globulin; GGT, *γ*-glutamyltransferase; IGM, immunoglobulin M; PLT, platelet count.

**Table 7:** Percentage of biochemical relative changes from baseline at 12 months.

%Change from baseline	13–15 mg/kg/d	18–22 mg/kg/d	*P* value
	(*n* = 36)	(*n* = 37)	
TB, *μ* mol/L	8.30 (−18.41, 25.40)	2.25 (12.84, 25.52)	0.860
ALP, IU/L	19.89 (3.68, 39.27)	21.16 (6.49, 34.16)	0.830
GGT, IU/L	−27.90 (−2.25, 55.16)	31.86 (−18.08, 42.67)	0.643
AST, IU/L	20.42 (−12.83, 31.50)	23.81 (7.31, 33.33)	0.277
ALT, IU/L	20.71 (−18.46, 39.65)	28.57 (9.57, 39.29)	0.351
ALB, g/L	−1.88 (−4.96, 6.93)	−0.44 (−3.52, 7.15)	0.354
GLB, g/L	2.23 (−2.37, 11.19)	4.05 (−1.70, 10.62)	0.544
PLT, 10^9^/L	11.67 (−0.49, 23.90)	1.72 (−18.03, 18.86)	0.075
IGM, mg/L	11.49 (−3.17, 20.14)	14.05 (8.18, 30.33)	0.120

*Note.* TB, total bilirubin; ALP, alkaline phosphatase; AST, aspartate aminotransferase; ALT, alanine aminotransferase; ALB, albumin; GLB, globulin; GGT, *γ*-glutamyltransferase; IGM, immunoglobulin M; PLT, platelet count.

**Table 8:** Adverse reactions through the course of the trial.

	13–15 mg/kg/d	18–22 mg/kg/d
	(*n* = 36)	(*n* = 37)
Diarrhea	0	2
Nausea and vomiting	1	1
Rash	1	1
High blood pressure	1	0

## Data Availability

No additional data are available.
